# Effectiveness of taking sequential mechanical ventilation in AECOPD with respiratory failure and risk factors for treatment failure

**DOI:** 10.3389/fmed.2026.1733746

**Published:** 2026-03-30

**Authors:** Ruijuan Xu, Haichao Liu, Yurong Liu, Congzheng Mao

**Affiliations:** Department of Respiratory and Critical Care Medicine, General Hospital of the Central Theater Command of PLA, Wuhan, China

**Keywords:** acute exacerbation, chronic obstructive pulmonary disease, respiratory failure, risk factors, sequential mechanical ventilation, treatment failure

## Abstract

**Background:**

To analyze the effect of sequential mechanical ventilation and the risk factors of treatment failure in patients with acute exacerbation of chronic obstructive pulmonary disease (AECOPD) with expiratory failure.

**Methods:**

This study retrospectively analyzed the clinical data of 125 patients with AECOPD and respiratory failure admitted to the Respiratory and Critical Care Medicine Department of the Central Theater Command General Hospital from January 2021 to December 2023 and observed the changes in arterial partial pressure of oxygen (PaO_2_), partial pressure of carbon dioxide (PaCO_2_), and heart rate (HR), respiration (RR), spirometry (FVC), as well as the changes in peak expiratory flow rate (PEF) before and after treatment. As well as the differences in the indexes of the first exertion expiratory volume (FEV1). Combined with the treatment outcome was divided into the treatment failure group the success group, and the logistic regression equation to determine the main factors of the failure of sequential ventilation treatment. Using ROC curve analysis to evaluate the predictive performance of logistic regression models, calculate the area under the curve (AUC) and its 95% confidence interval (CI).

**Results:**

PaO_2_, FVC, PEF, and FEV1 levels of the patients after treatment were increased compared with those before treatment, and the levels of PaCO_2_, HR, and RR were decreased (*p* < 0.05). Thirty cases were included in the failure group, and the remaining 95 cases were included in the success group. There were statistical differences in age, presence of medical diseases, acute physiology and chronic health evaluation II (APACHE II) score, PaO_2_, and PaCO_2_ levels between the two groups (*p* < 0.05). The ROC curve showed good discriminative ability, with an AUC of 0.864 (95% CI: 0.803–0.925, *p* < 0.05).

**Conclusion:**

Sequential ventilation therapy can improve blood gas indexes and lung function and stabilize vital signs in patients with AECOPD with expiratory failure. Moreover, age ≥60 years, presence of medical diseases, APACHE II score ≥19, PaO_2_ <55 mmHg, and PaCO_2_ ≥75 mmHg are risk factors for treatment failure.

## 1 Introduction

Chronic obstructive pulmonary disease (COPD), as a common lung disease, is characterized by incomplete and irreversible airway obstruction. The main symptoms of COPD are chronic cough and sputum production. The prevalence of COPD in the elderly population is around 9–10%, and it can affect the respiratory and circulatory functions of patients (1). A subset of COPD patients had ≥1.8 acute exacerbations per year. These episodes often trigger severe symptoms and respiratory failure, which in turn makes acute exacerbation of chronic obstructive pulmonary disease (AECOPD) the most common cause of death in this population, with respiratory failure further elevating mortality risk (2). ECOPD patients with respiratory failure frequently receive invasive ventilation to relieve symptoms and maintain airway patency (3). However, invasive ventilation is associated with long treatment durations and may lead to ventilator-associated pneumonia (4, 5). Therefore, sequential ventilation therapy has been proposed and applied in clinical practice to ensure ventilation efficacy and reduce the duration of invasive ventilation. However, treatment failure still occurs in some patients (6). Several studies have analyzed the reasons for treatment failure in AECOPD patients with respiratory failure, but there is no consensus on the specific influencing factors, and treatment failure remains an issue (7). Based on this, the purpose of this study is to analyze the effectiveness of sequential mechanical ventilation in patients with AECOPD with respiratory failure and identify the risk factors for treatment failure. This study summarizes the reasons for sequential ventilation therapy failure and aims to provide insights for improving future treatment success.

## 2 Patients and methods

### 2.1 Patients

This study retrospectively analyzed the clinical data of 125 patients with AECOPD and respiratory failure admitted to the Respiratory and Critical Care Medicine Department of the Central Theater Command General Hospital from January 2021 to December 2023. The diagnosis of COPD should be based on a comprehensive analysis of clinical manifestations, exposure history of risk factors, physical signs, and laboratory tests. A diagnosis of COPD should be considered clinically in any patient with dyspnea, chronic cough, or sputum production and a history of exposure to risk factors. and after inhaling bronchi relaxation first exertion expiratory volume (FEV1)/forced vital capacity (FVC) <70%.

The inclusion criteria were as follows: (1) Patients with a confirmed diagnosis of COPD were included. They presented with exacerbated cough, sputum production, and dyspnea. Patients without available spirometry were considered if they had high clinical suspicion of COPD based on medical history. These patients underwent targeted clinical review upon admission. The COPD diagnosis of all enrolled patients was jointly adjudicated and verified by two senior respiratory physicians. (2) Patients with type II respiratory failure were included. The diagnostic criteria were defined as arterial partial pressure of oxygen (PaO_2_) <60 mmHg and/or arterial partial pressure of carbon dioxide (PaCO_2_) >50 mmHg on arterial blood gas analysis. (3) Patients who received sequential mechanical ventilation; (4) patients with normal cognitive function; (5) patients with complete clinical data.

The exclusion criteria were as follows: (1) patients with other respiratory diseases such as tuberculosis or lung cancer; (2) patients who did not meet the indications for tracheal intubation and did not receive intermittent positive-pressure ventilation; (3) patients with immune deficiency diseases; (4) patients with major organ dysfunction such as liver or kidney dysfunction; (5) patients with severe infectious diseases such as sepsis; (6) patients who died during treatment; (7) patients with confirmed SARS-CoV-2 infection diagnosed.

**Figure 1:**
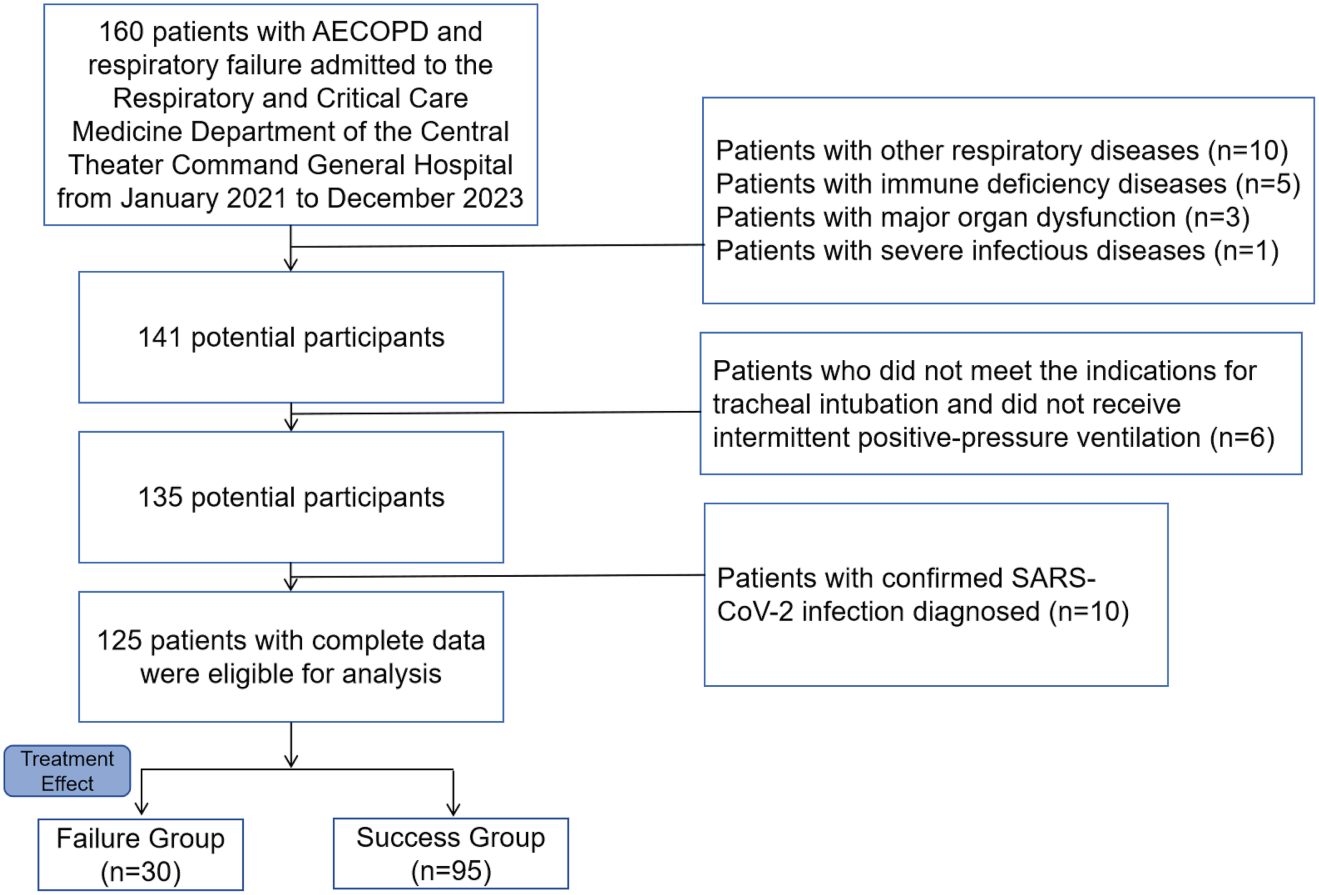
Inclusion and exclusion flowchart.

Informed consent was waived for this retrospective study due to the exclusive use of de-identified patient data, which posed no potential harm or impact on patient care. This waiver was approved by our hospital’s ethical review board and ethics committee, and adhered to regulatory and ethical guidelines for the Helsinki Declaration. Inclusion and exclusion flowchart see Figure 1.

### 2.2 Treatment methods

All patients received symptomatic treatment including antibiotics, bronchodilators and nebulized expectorants, and patients with heart failure were given diuretics, inotropic and vasodilator agents. All patients underwent sequential mechanical ventilation, with a median 5.5-h interval from admission to ventilation and an interquartile range of 4 to 7 h. Patients with severe respiratory distress received IMV within 3 to 4 h; moderate cases received pharmacotherapy plus non-invasive ventilation (NIV) and switched to invasive mechanical ventilation (IMV) at 8 h without improvement, with tracheal intubation performed by attending physicians according to routine clinical practice. IMV was delivered via tracheal intubation or tracheostomy using SIMV combined with PSV mode. Ventilator parameters included tidal volume 8 to 10 mL/kg and respiratory rate 12 to 20 breaths per minute, which were adjusted based on respiratory status and blood gas results. IMV was shifted to NIV when the PIC window was achieved. Post-extubation NIV was delivered via face mask with pressure support 8 to 15 cm H_2_O and positive end-expiratory pressure 3 to 5 cm H_2_O to maintain SpO_2_ no less than 90% and respiratory rate less than 30 breaths per minute. A 2-h spontaneous breathing trial with low-level pressure support 5 to 7 cm H_2_O preceded NIV weaning, and ventilation was stopped only after a successful trial. NIV failure or reintubation was defined as inability to sustain spontaneous breathing within 6 h post-extubation, severe respiratory distress, or the need for reintubation within 48 h.

### 2.3 Measurement parameters

Arterial blood gas analysis was performed to measure the levels of PaO_2_ and PaCO_2_ before and after treatment. Heart rate (HR) and respiratory rate (RR) were measured using a cardiac monitor. Pulmonary function testing using a lung function device (Germany Jaeger) was performed on AECOPD patients upon admission, recording the forced expiratory volume in the first second (FEV1), as well as the ratio of FEV1 to forced vital capacity (FEV1/FVC). Spirometry was performed to measure forced vital capacity (FVC), peak expiratory flow rate (PEF), and forced expiratory volume in 1 s (FEV1) before and 30 days after treatment. The patient’s medical records were reviewed to collect data on gender, age, body mass index, smoking history, alcohol consumption history, duration of COPD, presence of medical diseases, and APACHE II score.

### 2.4 Assessment of treatment outcome

Patients underwent assist-control ventilation after tracheal intubation via video laryngoscopy, with parameters adjusted to ensure sufficient oxygenation and ventilation. From the second day of mechanical ventilation, consciousness, oxygenation index, infection markers, Glasgow Coma Scale (GCS) score and PaCO_2_ were monitored to evaluate weaning eligibility. Sequential noninvasive ventilation was used after weaning criteria were met, with duration adjusted according to PaCO_2_ changes, and weaning was completed gradually under effective ventilation. Reintubation and conversion to invasive ventilation during sequential ventilation was defined as treatment failure (8).

### 2.5 Observation parameters

The changes in PaO_2_, PaCO_2_, HR, and RR before and 24 h after treatment, as well as the changes in FVC, PEF, and FEV1 before and 30 days after treatment, were observed. The patients were divided into the treatment failure group and the success group based on the treatment outcome. The differences in baseline data and clinical data between the two groups were observed, and the variables with differences in the univariate analysis were included in the logistic regression equation to determine the main factors contributing to treatment failure.

### 2.6 Statistical methods

SPSS 25.0 software was used for data analysis. Count data were expressed as *n* (%) and analyzed using the chi-square test or Fisher’s exact test. Normally distributed measurement data were expressed as mean ± standard deviation (SD) and analyzed using the *t*-test. Non-normally distributed measurement data were expressed as a median and interquartile range [M (P25, P75)] and analyzed using the Mann–Whitney *U* test. ROC curve analysis is used to evaluate the predictive performance of logistic regression models, calculating the area under the curve (AUC) and its 95% confidence interval (CI). The closer the AUC value is to 1, the better the predictive performance of the model. Variables with differences in the univariate analysis were included in the logistic regression equation. The criteria for variable selection were *p* = 0.05, and a *p* < 0.05 was considered statistically significant.

## 3 Results

### 3.1 Comparison of clinical data before and after treatment in all patients

Among 125 patients, 80 (64%) were male and 45 (36%) were female, with an average age of 68.3 ± 8.7 years. The average duration of COPD is 9.7 ± 3.4 years. Most patients (72%) had a history of smoking, and 38% had complications such as hypertension or diabetes. The acute physiology and chronic health assessment II (APACHE II) score range is 15 to 25, with an average score of 18.7 ± 2.9 (Table 1). Sequential mechanical ventilation significantly improved blood-gas parameters, pulmonary function, and vital signs in the 125 patients with AECOPD and respiratory failure. After treatment, arterial blood-gas analysis revealed a marked increase in PaO_2_ levels from 54.56 ± 9.14 mmHg to 76.58 ± 10.10 mmHg, while PaCO_2_ levels decreased significantly from 78.96 ± 10.52 mmHg to 50.30 ± 6.67 mmHg (*p* < 0.05). Similarly, pulmonary function tests demonstrated significant enhancements. The mean FVC increased from 2.44 ± 0.36 L to 3.05 ± 0.32 L, and the mean PEF rose from 3.30 ± 0.51 L/s to 5.04 ± 0.74 L/s, both showing statistically significant improvements (*p* < 0.05). The FEV1 improved from 1.76 ± 0.30 L to 2.52 ± 0.45 L. The HR decreased from 117.15 ± 9.52 beats/min to 87.40 ± 7.32 beats/min, while the RR was reduced from 26.50 ± 4.38 breaths/min to 16.62 ± 4.20 breaths/min (*p* < 0.05). Of the 125 patients included in the study, 30 cases were classified as treatment failure, accounting for 24.00% of the cohort, while the remaining 95 cases (76.00%) were classified as treatment success (Table 2). Data are presented as mean ± SD for normally distributed variables and median (IQR) for non-normally distributed variables.

**Table 1:** Baseline characteristics.

Characteristic	Participants (*n* = 125)
Gender	
Male	80 (64%)
Female	45 (36%)
Mean age (years)	68.3 ± 8.7
Duration of COPD (years)	9.7 ± 3.4
Smoking history	
Yes	90 (72%)
No	35 (28%)
Hypertension	31 (25%)
Diabetes	16 (13%)
APACHE II score	18.7 ± 2.9

Continuous variables are presented as mean ± standard deviation (SD); categorical variables are presented as *n* (%). Normality was assessed using the Shapiro–Wilk test.

**Table 2:** Comparison of clinical data before and after treatment in all patients.

Variable	Before treatment (*n* = 125)	After treatment (*n* = 125)	*t*	*p*-value
PaO_2_ (mmHg)	54.56 ± 9.14	76.58 ± 10.10	18.074	<0.001
PaCO_2_ (mmHg)	78.96 ± 10.52	50.30 ± 6.67	25.724	<0.001
HR (min)	117.15 ± 9.52	87.40 ± 7.32	27.697	<0.001
RR (min)	26.50 ± 4.38	16.62 ± 4.20	18.203	<0.001
FVC (L)	2.44 ± 0.36	3.05 ± 0.32	14.159	<0.001
PEF (mL/s)	3.30 ± 0.51	5.04 ± 0.74	21.646	<0.001
FEV1 (L)	1.76 ± 0.30	2.52 ± 0.45	15.711	<0.001

PaO_2_, arterial partial pressure of oxygen; PaCO_2_, arterial partial pressure of carbon dioxide; HR, heart rate; RR, respiratory rate; FVC, forced vital capacity; PEF, peak expiratory flow rate; FEV1, forced expiratory volume in the first second.

### 3.2 Univariate analysis of factors associated with treatment failure in patients with AECOPD and respiratory failure

Univariate analysis revealed no statistically significant differences in gender (*p* = 0.836), body mass index (*p* = 0.665), smoking history (*p* = 0.956), alcohol consumption history (*p* = 0.982), duration of COPD (*p* = 0.160), heart rate (*p* = 0.729), respiratory rate (*p* = 0.585), forced vital capacity (FVC, *p* = 0.568), peak expiratory flow (PEF, *p* = 0.679), and forced expiratory volume in 1 s (FEV1, *p* = 0.774) between the treatment failure group and the success group. However, statistically significant differences were observed in age, presence of medical diseases, APACHE II score, and blood-gas parameters (PaO_2_ and PaCO_2_) between the two groups.

To facilitate clinical interpretability in the univariate comparisons, continuous variables were additionally categorized into binary groups using clinically meaningful thresholds. Age was categorized at 60 years for clinical stratification, consistent with prior studies that defined elderly COPD/AECOPD populations as age ≥60 years (9). Severe hypoxemia was defined as PaO_2_ <55 mmHg based on guideline criteria (10). The APACHE II score was dichotomized using a threshold of 19, which has been used in prior AECOPD cohorts when assessing the risk of ventilatory treatment failure (11). Severe hypercapnia was defined as PaCO_2_ ≥75 mmHg as a high-risk feature reported in consensus guidance for acute hypercapnic respiratory failure managed with NIV (12).

Patients in the failure group were older, with a mean age of 73.65 ± 3.90 years compared to 66.45 ± 3.85 years in the success group (*p* < 0.05). The presence of internal diseases was significantly higher in the failure group (60.00% vs. 10.53%, *p* < 0.05). Differences in APACHE II scores were also significant, with 36.67% of patients in the failure group having scores ≥19, compared to 12.63% in the success group (*p* < 0.05). Arterial blood-gas analysis showed significant differences between the two groups. In the failure group, 56.67% of patients had PaO_2_ levels <55 mmHg, compared to 15.79% in the success group (*p* < 0.05). Similarly, 66.67% of patients in the failure group had PaCO_2_ levels ≥75 mmHg, compared to 26.32% in the success group (*p* < 0.05). These findings indicate significant disparities in age, comorbidity burden, disease severity (as reflected by APACHE II scores), and blood-gas parameters between the two groups (Table 3).

**Table 3:** Univariate analysis of factors associated with treatment failure in patients with AECOPD and respiratory failure.

Variable	Failure group (*n* = 30)	Success group (*n* = 95)	*χ*^2^/*t*/*Z*	*p*-value
Gender				
Male	18 (60.00)	59 (62.11)	0.043	0.836
Female	12 (40.00)	36 (37.89)		
Age (years)	73.65 ± 3.90	66.45 ± 3.85	8.902	<0.001
<60 years old	10 (33.33)	55 (57.89)	5.511	0.019
≥60 years old	20 (66.67)	40 (42.11)		
Body mass index (kg/m^2^)	22.75 ± 2.14	23.02 ± 3.18	0.434	0.665
Smoking history				
Yes	21 (70.00)	67 (70.53)	0.003	0.956
No	9 (30.00)	28 (29.47)		
Drinking history				
Yes	5 (16.67)	16 (16.84)	0.001	0.982
No	25 (83.33)	79 (83.16)		
COPD duration (years)	9.88 (2.15)	10.20 (2.17)	0.852	0.160
Whether having internal diseases				
Yes	18 (60.00)	10 (10.53)	32.105	<0.001
No	12 (40.00)	85 (89.47)		
APACHE II score (points)				
<19	19 (63.33)	83 (87.37)	31.428	<0.001
≥19	11 (36.67)	12 (12.63)		
PaO_2_ (mmHg)				
≥55	13 (43.33)	80 (84.21)	20.002	<0.001
<55	17 (56.67)	15 (15.79)		
PaCO_2_ (mmHg)				
≥75	20 (66.67)	25 (26.32)	16.112	<0.001
<75	10 (33.33)	70 (73.68)		
HR (times/min, $\bar{x}$ ± s)	118.25 ± 9.40	117.56 ± 9.52	0.347	0.729
RR (times/min, $\bar{x}$ ± s)	26.38 ± 4.22	25.90 ± 4.18	0.547	0.585
FVC [L, M (P25, P75)]	2.52 (2.10, 2.80)	2.60 (2.15, 2.78)	0.452	0.568
PEF [L/s, M (P25, P75)]	3.38 (2.86, 3.80)	3.46 (2.92, 3.75)	0.330	0.679
FEV1 [L, M (P25, P75)]	1.76 (1.50, 2.02)	1.80 (1.56, 2.00)	0.228	0.774

① AECOPD, acute exacerbation of chronic obstructive pulmonary disease; APACHE II, acute physiology and chronic health evaluation II; PaO_2_, arterial partial pressure of oxygen; PaCO_2_, arterial partial pressure of carbon dioxide; HR, heart rate; RR, respiratory rate; FVC, forced vital capacity; PEF, peak expiratory flow rate; FEV1, forced expiratory volume in the first second. ② Normal distribution: $\bar{x}$ ± s, *t*-test; categorical data: *n* (%), *χ*^2^-test; non-normal distribution: M (P25, P75), Mann–Whitney *U* test. ③ Cut-off values based on clinical practice/guidelines and ROC curve analysis. ④ Internal diseases: hypertension, diabetes, etc. ⑤ Two-tailed test, *p* < 0.05 was statistically significant.

### 3.3 Logistic regression analysis of factors associated with treatment failure in patients with AECOPD and respiratory failure

Based on the univariate analysis, variables with statistical significance and clinical relevance were included in the multivariable logistic regression model using the same categorization strategy as in Table 3 to facilitate clinical interpretation. Age was categorized as ≥60 years (assigned a value of 1) or <60 years (assigned a value of 0). The presence of medical diseases was recorded as 1 for patients with comorbidities such as hypertension or diabetes and 0 for those without. APACHE II scores were dichotomized, with scores ≥19 assigned a value of 1 and scores <19 assigned a value of 0. Similarly, arterial blood-gas parameters were categorized: PaO_2_ levels <55 mmHg were assigned a value of 1, while PaO_2_ levels ≥55 mmHg were assigned a value of 0. For PaCO_2_, levels ≥75 mmHg were recorded as 1, and levels <75 mmHg were recorded as 0 (Supplementary Table S1). These assignments facilitated the inclusion of these independent variables in subsequent multivariate logistic regression analysis to identify risk factors for treatment failure.

The logistic regression analysis revealed that ORs for age ≥60 years, presence of medical diseases, APACHE II score ≥19, PaO_2_ <55 mmHg, and PaCO_2_ ≥75 mmHg were 2.145 (95% CI: 1.456–2.528, *p* = 0.005), 1.495 (95% CI: 1.155–1.976, *p* = 0.027), 1.733 (95% CI: 1.288–2.739, *p* = 0.002), 1.528 (95% CI: 1.354–2.014, *p* = 0.003), and 1.446 (95% CI: 1.231–2.571, *p* < 0.05), respectively (Table 4). This suggests that age ≥60 years, presence of medical diseases, APACHE II score ≥19, PaO_2_ <55 mmHg, and PaCO_2_ ≥75 mmHg are risk factors for treatment failure in patients with AECOPD and respiratory failure.

**Table 4:** Logistic regression analysis of factors associated with treatment failure in patients with AECOPD and respiratory failure.

Influencing factors	Regression coefficient	Standard error	*z*	*χ* ^2^	*p*-value	OR value (95% CI)
Age ≥60 years old	0.763	0.259	2.946	8.680	0.003	2.145 (1.456–3.158)
Having internal diseases	0.401	0.172	2.331	5.434	0.020	1.495 (1.155–1.938)
APACHE II score ≥19	0.549	0.176	3.120	9.734	0.002	1.733 (1.288–2.338)
PaO_2_ <55 mmHg	0.423	0.131	3.229	10.426	0.001	1.528 (1.354–1.725)
PaCO_2_ ≥75 mmHg	0.369	0.105	3.514	12.348	<0.001	1.446 (1.231–1.698)

### 3.4 ROC curve analysis for logistic regression model

**Figure 2:**
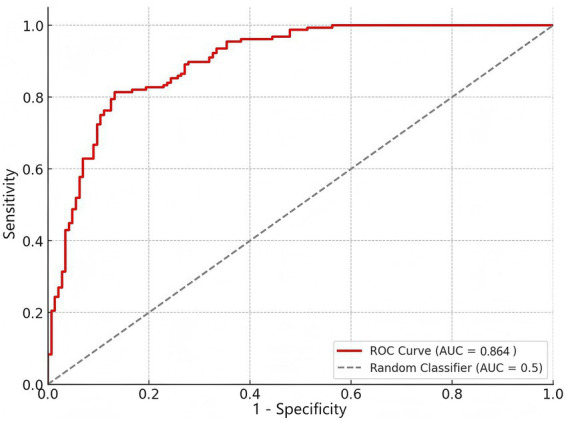
ROC curve analysis for logistic regression model. The ROC curve demonstrates the predictive performance of the logistic regression model for identifying treatment failure in patients with AECOPD and respiratory failure. The area under the curve (AUC) is 0.864, indicating good discriminatory ability of the model. The dashed line represents the performance of a random classifier (AUC = 0.5).

To evaluate the predictive performance of logistic regression model in identifying treatment failure in AECOPD patients with respiratory failure, ROC curve analysis was conducted. Cut-off points for continuous variables were based on clinical guidelines or previous studies (10, 13, 14). This model combines five independent risk factors identified from multivariate logistic regression analysis, including age ≥60 years, presence of internal medicine, APACHE II score ≥19, PaO_2_ <55 mmHg, and PaCO_2_ ≥75 mmHg. The ROC curve showed good discriminative ability, with an AUC of 0.864 (95% CI: 0.803–0.925, *p* < 0.05). The optimal critical value for predicting treatment failure is 0.58, with a sensitivity of 83.3% and a specificity of 78.9% (Figure 2).

## 4 Discussion

COPD is one of the common respiratory diseases, which is caused by both individual and environmental factors. Individual factors include age, gender, and ischemic hypoxia caused by diseases or developmental abnormalities, while environmental factors include smoking, respiratory infections, and environmental pollution leading to airway damage and inflammation (15). Previous study has found that sequential mechanical ventilation is a new ventilation method for AECOPD with respiratory failure (16). Although multiple studies have investigated predictors of ventilatory treatment failure in AECOPD complicated by respiratory failure, the reported risk factors and prediction models vary substantially across studies due to heterogeneity in patient populations, outcome definitions, and analytic approaches; therefore, the clinical applicability of existing models remains to be further validated (17–19). Therefore, how to reduce the risk of sequential ventilation treatment failure has gradually attracted clinical attention. The risk factors of sequential therapy need to be recognized by the public, to increase the success rate of sequential therapy and reduce the suffering of patients.

Studies have shown that sequential mechanical ventilation can make patients PO_2_ rise and decline in PCO_2_, which is consistent with the results of our study (20). This reflects that AECOPD with blood gas index, and pulmonary function in patients with respiratory failure, can pass sequence through improvement in gas treatment. Firstly, sequential invasive-to-noninvasive ventilation may improve gas exchange in AECOPD with respiratory failure by supporting oxygenation and enhancing alveolar ventilation, thereby reducing hypercapnia (3, 13). Titration of ventilatory settings (e.g., tidal volume, respiratory rate, and pressure support) can modify minute ventilation and ventilation efficiency, which may translate into improved arterial blood gas parameters (increased PaO_2_ and decreased PaCO_2_) (21, 22). Secondly, for patients with respiratory function impairment, reasonable ventilation support during sequential ventilation can relieve the fatigue of respiratory muscles. By adjusting ventilation parameters, complications such as alveolar collapse or excessive lung expansion can be prevented, and lung function recovery can be accelerated (23). Ventilation support during sequential ventilation helps maintain stable respiratory function and ensure normal oxygenation. It also avoids respiratory arrest or excessive ventilation to keep vital signs stable. This may be related to improved lung function in patients with a direct relationship,

AECOPD patients admitted to the ICU face a high risk of in-hospital death. An elevated APACHE II score is associated with increased ventilation treatment failure risk, as a higher score may indicate more severe illness, greater difficulty in sequential ventilation management, and potential impairment of post-extubation spontaneous breathing recovery. Li et al. reported the APACHE II score as a prognostic tool with an AUC of 0.675 (95% CI: 0.620–0.730, *p* < 0.001) (24). In contrast, this study’s logistic regression model incorporating the APACHE II score achieved an AUC of 0.864, showing better accuracy in distinguishing treatment outcomes and ensuring cross-cohort reliability.

This study still has limitations: We selected AECOPD patients who received ventilation treatment in our hospital within a specific time range, which may limit the generalizability of the sample. This study used a retrospective cohort study design, which cannot eliminate potential confounding factors and information bias, but we collected as much information as possible for both groups. To offset the impact of confounding factors and information. This study was conducted in a specific medical institution, and the unique characteristics of this institution may limit the generalizability of the research results to other medical institutions with different backgrounds and clinical practices. The analysis of influencing factors did not include nutritional status, blood lactate, and serum inflammatory factors, this research will focus on the patient’s lung signs, so smaller than its impact on nutritional status, but we analyzed other influencing factors as much as possible. Furthermore, this study excluded patients with COVID-19 infection to focus solely on AECOPD-related respiratory failure. However, the broader healthcare context during the pandemic, such as strained resources or delayed hospital visits, may still have influenced the results. Future research can make up for these limitations through more refined designs and larger samples in multicenter studies. Despite these limitations, this study provides substantial support for reducing the failure rate of treatment in patients with AECOPD with respiratory failure. This simple and feasible clinical intervention is expected to be widely used in practical work to improve treatment outcomes in patients with AECOPD with respiratory failure.

## 5 Conclusion

Through this clinical retrospective analysis, we have gained a deeper understanding of the clinical characteristics of AECOPD and the improvement in blood gas indexes and lung function of AECOPD patients with respiratory failure after sequential ventilation treatment. The study shows that sequential ventilation can improve blood gas indexes and lung function and stabilize vital signs in AECOPD patients with respiratory failure. Age ≥60 years, presence of medical diseases, APACHE II score ≥19, PaO_2_ <55 mmHg, and PaCO_2_ ≥75 mmHg are risk factors for treatment failure. In summary, this study provides a reference for the sequential ventilation treatment of the disease, further enhances quality of medical services, improve the treatment effect and quality of life of patients, and offers valuable experience and reference for future research on such diseases.

## Data Availability

The original contributions presented in the study are included in the article/Supplementary material, further inquiries can be directed to the corresponding author.
