# Supplementary material for: Effectiveness of taking sequential mechanical ventilation in AECOPD with respiratory failure and risk factors for treatment failure
Source: Front Med (Lausanne). 2026 Mar 30;13:1733746. doi: 10.3389/fmed.2026.1733746 (PMC13071063; doi:10.3389/fmed.2026.1733746)
Supplement: Supplementary file 1 [file Table_1.DOCX]

**Table S1.** Assignment of Independent Variables

| Independent variable | Assignment |
| --- | --- |
| Age | For patients ≥60 years old, the value is 1; for patients <60 years old, the value is 0. |
| Whether having internal diseases | For patients with internal diseases, the value is 1; for patients without internal diseases, the value is 0. |
| APACHE II score | For patients ≥19 points, the value is 1; for patients <19 points, the value is 0. |
| PaO_2_ | For patients <55mmHg, the value is 1; for patients ≥55mmHg, the value is 0. |
| PaCO_2_ | For patients ≥75mmHg, the value is 1; for patients <75mmHg, the value is 0. |
